# Association of sarcopenia with the long‐term risk of atrial fibrillation: A prospective cohort study

**DOI:** 10.1111/acel.14198

**Published:** 2024-05-13

**Authors:** Yiyang Tang, Zhenghui Liu, Qin Chen, Mukamengjiang Juaiti, Zaixin Yu, Benhui Liang, Lihuang Zha

**Affiliations:** ^1^ Department of Cardiology Xiangya Hospital, Central South University Changsha Hunan China; ^2^ Department of Neurology Xiangya Hospital, Central South University Changsha Hunan China; ^3^ National Clinical Research Center for Geriatric Disorders Xiangya Hospital, Central South University Changsha Hunan China

**Keywords:** atrial fibrillation, prospective cohort, sarcopenia, UK biobank

## Abstract

The relationship between sarcopenia and the long‐term risk of atrial fibrillation (AF) remains unclear. This study recruited a large prospective Caucasian cohort from the UK Biobank. Participants were assessed at baseline with handgrip strength and muscle mass and were categorized into groups of non‐sarcopenia, probable sarcopenia, and confirmed sarcopenia. Kaplan–Meier method and Cox proportional hazards model were used to explore the association between sarcopenia and the incidence of AF. The genetic predisposition of AF was assessed by polygenic risk score. Sensitivity analyses were performed to validate the results. A total of 384,433 participants with a median age of 58 years and 54.3% women were enrolled in this study. There were 24,007 cases of new‐onset AF over a median follow‐up of 12.56 years. The groups of non‐sarcopenia, probable sarcopenia, and confirmed sarcopenia accounted for 22,290 (6.1%), 1665 (9.2%), and 52 (11.9%) cases, respectively. Compared with the non‐sarcopenia group, participants with probable sarcopenia or confirmed sarcopenia had an 8% (95% CI, 1.03–1.14) or 61% (95% CI, 1.23–2.12) higher risk of AF incidence. The findings remained robust in multiple sensitivity analyses, such as subgroup analysis and further adjustment of genetic predisposition. Notably, the association between sarcopenia and a high AF risk was more pronounced in younger participants, women, and those with valvular heart disease. In conclusion, sarcopenia was associated with a high long‐term risk of AF in Caucasians, supporting sarcopenia as a new independent risk factor of AF.

## Graphical abstract

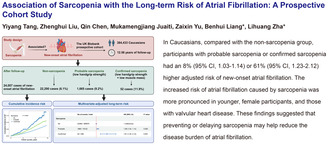

**Abbreviations**
AF: atrial fibrillation
ALT: alanine aminotransferase
CI: confidence interval
COPD: chronic obstructive pulmonary disease
HR: hazard ratio
MET: metabolic equivalent task
PRS: polygenic risk score

## 1 INTRODUCTION

Atrial fibrillation (AF) is the most common sustained arrhythmia, affecting an estimated 2% to 4% of adults (Hindricks et al., 2021). As the global population ages, it has been predicted that by 2050, 6–12 million individuals in the United States and, by 2060, 17.9 million individuals in Europe will suffer this condition (Krijthe et al., 2013; Patel et al., 2014). As a result, AF markedly heightens the risks of death, stroke, heart failure, cognitive impairment, and dementia, which substantially diminishes the life quality of the patients (Chung et al., 2020). Hence, identifying the risk factors of AF and providing early protective measures for high‐risk individuals are of paramount significance in alleviating both personal sufferings and national socioeconomic burdens.

Sarcopenia is a progressive skeletal muscle disorder with age‐dependent morbidity characteristics and ethnic differences (Cruz‐Jentoft & Sayer, 2019). The evaluation criteria of sarcopenia were established in Asian and Caucasian populations, respectively. In brief, the Asian consensus focuses on the loss of muscle mass with age in the sarcopenia definition (Chen et al., 2020). The European consensus highlights impaired muscle function in terms of practicality, but it also indicates that confirmed sarcopenia must include low muscle quantity or quality (Cruz‐Jentoft et al., 2019). The recommended cut‐off points for sarcopenia tests also differ between the two consensuses. Sarcopenia was confirmed to be closely related to heightened risks of fractures, osteoporosis, frequent hospitalization, declined quality of life, and mortality (Jauffret et al., 2023; Petermann‐Rocha et al., 2021; Wei et al., 2023). Recently, there has been increasing interest in understanding sarcopenia in patients with cardiovascular diseases. The interest started in the field of heart failure and gradually extended to other areas, including AF (Damluji et al., 2023).

There is a potential relevance between sarcopenia and AF. Compared with participants without AF, AF patients had lower handgrip strength, slower walking speed, and reduced proportion of skeletal muscle to body weight (Anaszewicz et al., 2019; Yang et al., 2020). Several cross‐sectional studies demonstrated that the presence of sarcopenia was associated with a high incidence of AF after multivariate adjustments (Shim et al., 2023; Xia et al., 2021). However, in a cohort study with 2 years of follow‐up, the risk of new‐onset AF was not increased in patients with sarcopenia (Shim et al., 2023). Further prospective studies are required to elucidate their relationship. Additionally, current related studies predominantly focused on Asian populations. It is imperative to investigate this association among Caucasians.

This study employed a UK Biobank cohort with 384,433 participants and a median follow‐up duration of 12.56 years to investigate the association between sarcopenia and the long‐term risk of AF in Caucasians. It aims to provide epidemiological evidence for identifying a new risk factor of AF and contribute to optimizing its early prevention.

## 2 METHODS

### 2.1 Study participants

Figure S1 demonstrated an overview of the study design. The data of this study were extracted from the UK Biobank under Application 107175 (Sudlow et al., 2015). In brief, this project recruited over half a million participants aged 40–69, from 22 regions in the United Kingdom from 2006 to 2010. It received ethics approval from the North West Multi‐Centre Research Ethics Committee. The data of self‐reported health behaviors, physical examinations, and biological sample analyses were collected and followed up for a long period. This study conformed to the Strengthening the Reporting of Observational Studies in Epidemiology (STROBE) reporting guideline.

Only Caucasian participants were included. Participants with AF at baseline (*n* = 8128) were excluded, followed by participants with missing data on handgrip strength (*n* = 1555), muscle mass (*n* = 7230), height (*n* = 98), and covariates (*n* = 71,119). Finally, 384,433 participants were included in the main analysis.

### 2.2 Ascertainment of the outcome

The follow‐up period commenced on the day that the study participants completed the baseline survey and extended until the onset of AF, mortality, loss to follow‐up, or the termination of follow‐up, whichever event transpired first. The end dates for follow‐up were September 30, 2021, for England and Wales, and October 31, 2021, for Scotland. Diagnosis of AF was determined through death registration, primary care, and hospital admission records. The data were extracted from the diagnosis codes following the International Classification of Diseases, Tenth Revision (Table S1). These data was provided by patients and collected by the NHS as part of their care and support.

### 2.3 Assessment of sarcopenia

The assessment of sarcopenia adhered to the guidelines established by the European Working Group on Sarcopenia in Older People (Cruz‐Jentoft et al., 2019), involving muscle strength, muscle quantity, and physical performance. According to the criteria delineated in Table S2, participants only exhibiting low handgrip strength were categorized as having probable sarcopenia; participants exhibiting both low handgrip strength and low muscle mass were classified as having confirmed sarcopenia; and those exhibiting low handgrip strength, low muscle mass, and poor physical performance were identified as severe sarcopenia cases. It should be noted that the limited cases of severe sarcopenia (*n* = 100) were included in the group of confirmed sarcopenia for subsequent analyses.

Handgrip strength measurements of both hands were evaluated using a Jamar J00105 hydraulic hand dynamometer. The higher value of the two measurements was taken as an individual's handgrip strength. The values <16 kg for women and <27 kg for men were identified as low handgrip strength. Muscle mass was assessed through appendicular lean soft tissue/the square of height, which followed the equation: appendicular lean soft tissue = (0.958 × appendicular fat − free mass) − (0.166 × S) − 0.308, where fat‐free mass was obtained by bioelectrical impedance analysis and S was 0 for women and 1 for men. This equation and the reference value were established based on a dual X‐ray absorptiometry body composition scan of 4350 participants in the UK Biobank (Dodds et al., 2020). Women with the value <5.30 kg/m^2^ and men with the value <6.95 kg/m^2^ were considered to have low muscle mass (Kiss et al., 2023). Lastly, the poor physical performance of participants was determined by self‐reported slow walking pace in their daily lives.

### 2.4 Assessment of covariates

Multiple covariates were included in this study, encompassing demographic characteristics (age and gender), socioeconomic factors (education level and Townsend deprivation index), lifestyle factors (alcohol consumption, smoking, physical inactivity, dietary pattern, and body mass index), laboratory tests (C‐reactive protein, alanine aminotransferase, creatinine, and albumin), comorbidities (heart failure, valvular heart disease, coronary heart disease, chronic obstructive pulmonary disease, autoimmune diseases, hyperthyroidism, diabetes, hypertension, and hyperlipidemia), and major surgeries. The codes for retrieving these covariates in the UK Biobank were presented in Table S1. The information on demographic characteristics, socioeconomic factors, lifestyle factors, and major surgeries was collected by trained program staff through questionnaires at baseline. Townsend deprivation index was a continuous variable provided by the UK Biobank, with higher values reflecting lower socioeconomic status. Physical inactivity was assessed using Metabolic Equivalent Task (MET) minutes per week, encompassing walking, moderate, and vigorous activities. It was defined as less than 500 MET minutes per week (Elliott et al., 2020). Dietary data included various food items (vegetables, fruits, fish, processed meats, unprocessed meats, whole grains, and refined grains). As shown in Table S3, participants meeting each recommended dietary criterion were assigned one score (Mozaffarian, 2016). Scores ranging from 0 to 2, 3 to 5, and 6 to 7 indicated poor, moderate, and healthy dietary patterns, respectively. Body mass index was calculated by dividing weight by the square of height. The term major surgeries was a binary variable according to whether participants received major surgeries defined by the UK Biobank. The data of laboratory tests were recorded when participants received physical examinations. The diagnosis of most comorbidities conformed to the International Classification of Diseases, Tenth Revision. However, hypertension was defined as self‐reported diagnosis, antihypertensive medication intake, or measured blood pressure ≥140/90 mmHg. Diabetes was defined as self‐reported diagnosis, hypoglycemic medication intake, or a glycated hemoglobin level exceeding 6.5%. Hyperlipidemia was defined as cholesterol‐lowering medication intake or a low‐density lipoprotein level surpassing 4.0 mmol/L.

### 2.5 Statistical analyses

To describe baseline characteristics, categorical variables were presented as cases (percentages), with intergroup comparison using the Chi‐square test. Continuous variables that did not conform to normal distribution were expressed as medians (interquartile ranges) and were compared by the Mann–Whitney *U* test or Kruskal–Wallis *H* test.

The cumulative incidence of AF in the non‐sarcopenia, probable sarcopenia, and confirmed sarcopenia groups was illustrated using the Kaplan–Meier method, and the Log‐rank test was conducted to compare the differences between the groups. The Cox proportional hazards model was used to further investigate the association between sarcopenia and the incidence of AF. The results were presented as hazard ratio (HR) and 95% confidence interval (95% CI). In the Schoenfeld residuals test, no violations of the proportional hazards assumption were observed in the regression analysis. Multivariate model 1 was adjusted for age, gender (man or woman), education level (college, high school, middle school, or others), and the values of Townsend deprivation index and body mass index. Multivariate model 2 was further adjusted for the status of drinking and smoking (never, previous, or current), physical inactivity (yes or no), dietary pattern (poor, moderate, or healthy), the values of laboratory tests, the comorbidities (yes or no), and the major surgeries (yes or no). The association between handgrip strength or muscle mass and AF incidence was also investigated using the above analyses.

Multiple sensitivity analyses were conducted to ensure the robustness of the results. First, we performed subgroup analysis stratified by age (<65 or ≥ 65 years), gender (men or women), education level (college, high school, middle school, or other), Townsend deprivation index (quintile 1, quintile 2–4, or quintile 5), body mass index (<25 or ≥ 25), the status of drinking and smoking (never, previous, or current), physical inactivity (yes or no), dietary pattern (poor, moderate, or healthy), and comorbidities (yes or no). Second, we repeated analyses after imputing missing covariates through multiple imputation. Third, we limited the diagnosis of AF to hospital‐recorded AF to reduce potential misclassification bias. Fourth, we excluded participants who developed AF within the first 2 years of follow‐up to diminish the influence of reverse causality. Fifth, we reassessed muscle mass through an alternative criterion called skeletal muscle index, which was presented as dividing skeletal muscle mass by the square of height. The value of skeletal muscle mass was determined by the Janssen equation (Janssen et al., 2000). Participants with skeletal muscle index <5.5 kg/m^2^ for women and <7.0 kg/m^2^ for men were identified as having low muscle mass. Sixth, we established multivariate model 3 by further adjusting for medication intake (antiplatelet, antihypertensive, antidiabetic, and cholesterol‐lowering drugs) based on the model 2. Seventh, a sub‐distribution hazard model for competing risk was applied, regarding death from other causes as the competing risk.

Lastly, we explored the impact of genetic predisposition on the association of sarcopenia with AF by extracting the polygenic risk score (PRS) for AF in the UK Biobank PRS release (Thompson et al., 2022). The association between PRS levels and AF incidence was examined to ensure the effectiveness of PRS. Next, we added PRS as an additional covariate in the multivariate Cox models and stratification analysis, and then we repeated the analyses.

All statistical analyses were performed in R (version 4.2.2). A two‐tailed *p* < 0.05 was considered statistically significant.

## 3 RESULTS

### 3.1 Baseline characteristics

The process of inclusion and exclusion is illustrated in Figure S2. A total of 384,433 Caucasian participants with a median age of 58 years (interquartile range, 50–63) and 208,923 (54.3%) women were enrolled in this study. Table 1 showed the baseline characteristics of participants. Within the cohort, 365,875 (95.2%) participants were classified as having non‐sarcopenia, 18,122 (4.7%) as cases of probable sarcopenia, and 436 (0.1%) as cases of confirmed sarcopenia. In contrast to participants without sarcopenia, those with probable and confirmed sarcopenia exhibited older ages. Participants with confirmed sarcopenia were more likely to be male, current smokers, and to have physical inactivity, poor dietary patterns, and chronic diseases including heart failure and chronic obstructive pulmonary disease.

**TABLE 1:** Baseline characteristics of participants categorized by non‐sarcopenia, probable sarcopenia, and confirmed sarcopenia.

Variables	Total population	Sarcopenia			*p‐*Value
		Non	Probable	Confirmed	
*N* (%)	384,433	365,875 (95.2)	18,122 (4.7)	436 (0.1)	
Age (years)	58.00 [50.00, 63.00]	58.00 [50.00, 63.00]	62.00 [56.00, 66.00]	63.00 [59.00, 67.00]	<0.001
Women, *n* (%)	208,923 (54.3)	197,219 (53.9)	11,654 (64.3)	50 (11.5)	<0.001
Education level, *n* (%)					<0.001
College	54,662 (14.2)	52,753 (14.4)	1864 (10.3)	45 (10.3)	
High school	17,176 (4.5)	16,465 (4.5)	689 (3.8)	22 (5.0)	
Middle school	178,177 (46.3)	171,365 (46.8)	6668 (36.8)	144 (33.0)	
Others	134,418 (35.0)	125,292 (34.2)	8901 (49.1)	225 (51.6)	
Townsend deprivation index	−2.27 [−3.70, 0.22]	−2.31 [−3.72, 0.16]	−1.58 [−3.30, 1.47]	−0.86 [−2.96, 3.48]	<0.001
Body mass index	26.70 [24.10, 29.80]	26.70 [24.10, 29.80]	27.50 [24.60, 31.10]	20.50 [18.98, 21.80]	<0.001
Drinking status, *n* (%)					<0.001
Never	12,603 (3.3)	11,396 (3.1)	1187 (6.6)	20 (4.6)	
Previous	13,005 (3.4)	11,747 (3.2)	1218 (6.7)	40 (9.2)	
Current	358,825 (93.3)	342,732 (93.7)	15,717 (86.7)	376 (86.2)	
Smoking status, *n* (%)					<0.001
Never	208,546 (54.2)	198,723 (54.3)	9646 (53.2)	177 (40.6)	
Previous	135,600 (35.3)	128,910 (35.2)	6564 (36.2)	126 (28.9)	
Current	40,287 (10.5)	38,242 (10.5)	1912 (10.6)	133 (30.5)	
Physical inactivity, *n* (%)	45,117 (11.7)	42,274 (11.6)	2765 (15.3)	78 (17.9)	<0.001
Dietary pattern					<0.001
Healthy	30,031 (7.8)	28,772 (7.9)	1245 (6.9)	14 (3.2)	
Moderate	192,688 (50.1)	184,034 (50.3)	8502 (46.9)	152 (34.9)	
Poor	73,622 (19.2)	69,960 (19.1)	3549 (19.6)	113 (25.9)	
Laboratory tests					
C‐reactive protein, mg/L	1.32 [0.65, 2.74]	1.30 [0.65, 2.68]	1.93 [0.93, 4.06]	1.27 [0.52, 4.15]	<0.001
ALT, U/L	20.10 [15.39, 27.33]	20.11 [15.40, 27.34]	20.06 [15.30, 27.25]	17.66 [13.62, 23.41]	<0.001
Albumin, g/L	45.23 [43.54, 46.95]	45.26 [43.57, 46.98]	44.62 [42.84, 46.41]	44.50 [42.75, 46.57]	<0.001
Creatinine, μmol/L	70.30 [61.40, 80.70]	70.40 [61.50, 80.80]	67.30 [58.70, 77.80]	71.45 [61.68, 81.33]	<0.001
Comorbidities, *n* (%)					
Heart failure	1210 (0.3)	1057 (0.3)	147 (0.8)	6 (1.4)	<0.001
Valvular heart disease	1799 (0.5)	1661 (0.5)	136 (0.8)	2 (0.5)	<0.001
Coronary heart disease	19,037 (5.0)	17,300 (4.7)	1699 (9.4)	38 (8.7)	<0.001
COPD	7270 (1.9)	6472 (1.8)	748 (4.1)	50 (11.5)	<0.001
Hyperthyroidism	4021 (1.0)	3730 (1.0)	288 (1.6)	3 (0.7)	<0.001
Hyperlipidemia	177,114 (46.1)	166,959 (45.6)	9991 (55.1)	164 (37.6)	<0.001
Hypertension	208,793 (54.3)	197,244 (53.9)	11,320 (62.5)	229 (52.5)	<0.001
Diabetes mellitus	21,113 (5.5)	19,129 (5.2)	1959 (10.8)	25 (5.7)	<0.001
Autoimmune diseases	10,375 (2.7)	8515 (2.3)	1820 (10.0)	40 (9.2)	<0.001
Major surgeries	249,380 (64.9)	236,044 (64.5)	13,039 (72.0)	297 (68.1)	<0.001
Outcome, *n* (%)					
Atrial fibrillation	24,007 (6.2)	22,290 (6.1)	1665 (9.2)	52 (11.9)	<0.001

*Note*: Data were presented as median [interquartile ranges], or *n* (%).

Abbreviations: ALT, alanine aminotransferase; COPD, chronic obstructive pulmonary disease.

### 3.2 Association between sarcopenia and the long‐term risk of AF

**FIGURE 1:**
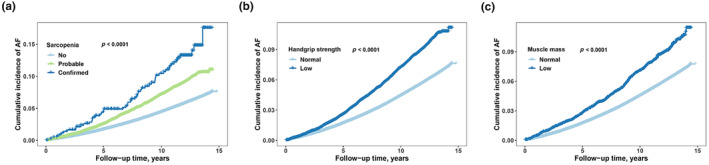
The cumulative incidence of atrial fibrillation (AF) with follow‐up time in groups categorized by sarcopenia (a), handgrip strength (b), and muscle mass (c). (a) The light blue, green, and dark blue curves represent non‐sarcopenia, probable, and confirmed sarcopenia groups, respectively; (b) The light blue and dark blue curves represent control and low handgrip strength groups, respectively; (c) The light blue and dark blue curves represent control and low muscle mass groups, respectively.

During a median follow‐up of 12.56 years, a total of 24,007 participants developed new‐onset AF. There were 22,290 (6.1%), 1665 (9.2%), and 52 cases (11.9%) in the groups of non‐sarcopenia, probable sarcopenia, and confirmed sarcopenia, respectively (Table 1). Similarly, the cumulative incidence of AF was higher in the probable sarcopenia group compared to the non‐sarcopenia group and appreciably higher in the confirmed sarcopenia group (Figure 1; *p* for log‐rank <0.001). Participants with low handgrip strength or low muscle mass also exhibited a higher cumulative incidence of AF than the control group (both *p* for log‐rank <0.001).

**FIGURE 2:**
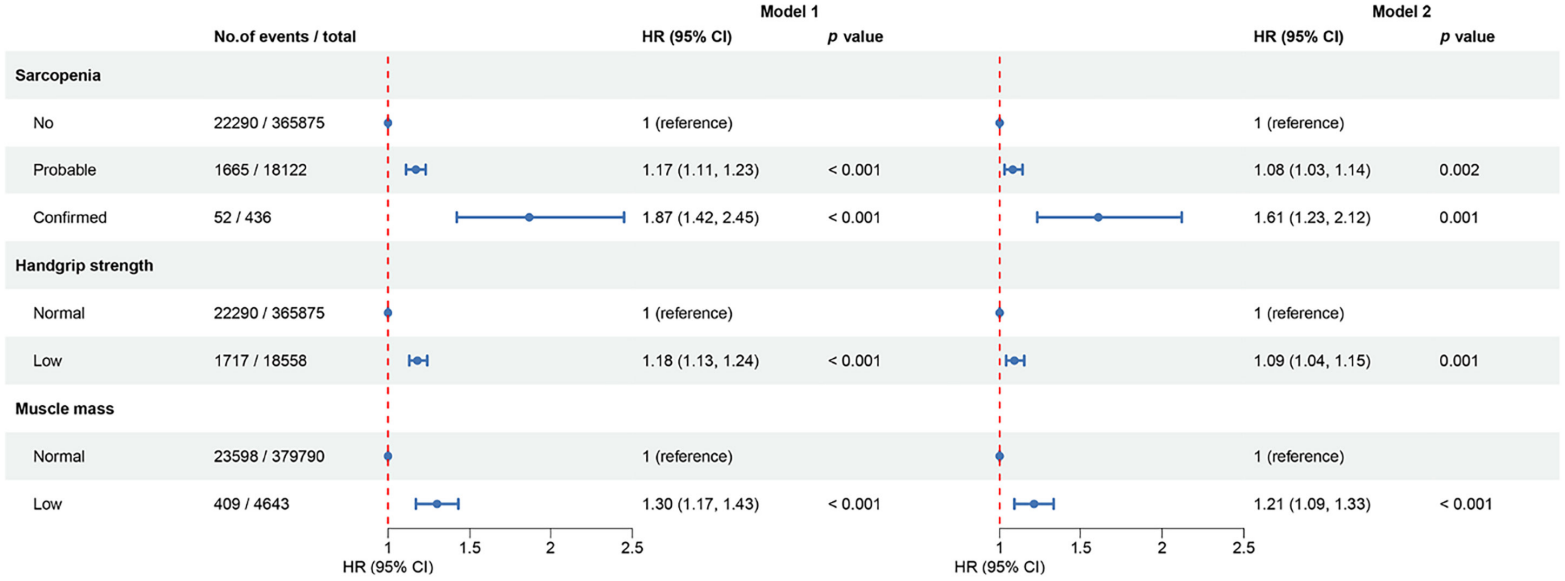
Association of sarcopenia and its components with the incidence of atrial fibrillation in multivariate Cox regression models. Model 1 was adjusted for age, gender, education level, Townsend deprivation index, and body mass index. Based on model 1, model 2 was further adjusted for the status of drinking and smoking, physical inactivity, dietary pattern, laboratory tests (C‐reactive protein, alanine aminotransferase, creatinine, and albumin), comorbidities (heart failure, valvular heart disease, coronary heart disease, chronic obstructive pulmonary disease, autoimmune diseases, hyperthyroidism, diabetes, hypertension, and hyperlipidemia), and major surgeries.

To further validate our findings, Cox proportional hazards models were established, with the non‐sarcopenia group serving as the reference (Figure 2). In multivariate model 1, HRs for AF incidence in the probable sarcopenia and confirmed sarcopenia groups were 1.17 (95% CI, 1.11–1.23) and 1.87 (95% CI, 1.42–2.45), respectively. After further adjusting for lifestyle factors, laboratory tests, comorbidities, and major surgeries in model 2, the HRs in the two groups were 1.08 (95% CI, 1.03–1.14) and 1.61 (95% CI, 1.23–2.12), respectively. Additionally, the HR for AF incidence in participants with low handgrip strength or low muscle mass was 1.09 (95% CI, 1.04–1.15) or 1.21 (95% CI, 1.09–1.33) when compared with control participants in the model 2.

### 3.3 Sensitivity analyses

In subgroup analysis, an increased AF risk of participants with confirmed sarcopenia was presented in all subgroups (Table 2). The HR of confirmed sarcopenia in the subgroup of hyperthyroidism cannot be correctly estimated because of too limited cases. The association between probable sarcopenia and the high incidence of AF was also significant, except for several subgroups, such as participants with previous alcohol consumption. Among these subgroups, AF risk was appreciably higher in women (*p* for interaction = 0.029), participants <65 years old (*p* for interaction = 0.002), or participants with valvular heart disease (*p* for interaction = 0.018) than in men, older participants, or those without valvular heart disease, respectively. No significant interaction was observed in other subgroups. The association of low handgrip strength or low muscle mass with the high incidence of AF was also not changed (Table S4). Furthermore, the association between sarcopenia and AF risk was tested by repeating the analyses after imputation of missing covariates (Figure S3), only including hospital‐recorded AF (Figure S4), excluding new‐onset AF within the first 2 years of follow‐up (Figure S5), defining sarcopenia based on an alternative criterion (Figure S6), further adjusting for medication intake on the model 2 (Figure S7), or incorporating a competing risk model (Figure S8). The conclusion remained robust.

**TABLE 2:** Subgroup analysis of the association between sarcopenia and the incidence of atrial fibrillation.

Subgroup	*N*	Sarcopenia			*p* for interaction
		Non	Probable	Confirmed	
Gender					0.029
Women	208,923	1 (reference)	1.05 (0.98, 1.13)	3.99 (1.99, 8.00)	
Men	175,510	1 (reference)	1.09 (1.02, 1.17)	1.56 (1.16, 2.10)	
Age					0.002
<65	310,838	1 (reference)	1.21 (1.13, 1.31)	2.17 (1.45, 3.25)	
≥65	73,595	1 (reference)	1.09 (1.02, 1.17)	1.48 (1.02, 2.15)	
Townsend deprivation index					0.778
Low	77,441	1 (reference)	1.08 (0.94, 1.23)	1.85 (0.92, 3.71)	
Moderate	230,200	1 (reference)	1.09 (1.01, 1.16)	1.34 (0.88, 2.04)	
High	76,792	1 (reference)	1.09 (0.99, 1.19)	1.92 (1.26, 2.94)	
Education					0.212
College	54,662	1 (reference)	1.12 (0.96, 1.32)	1.79 (0.74, 4.32)	
High school	17,176	1 (reference)	1.16 (0.89, 1.52)	4.50 (1.98, 10.20)	
Middle school	178,177	1 (reference)	1.01 (0.92, 1.11)	1.70 (1.02, 2.82)	
Others	134,418	1 (reference)	1.10 (1.03, 1.18)	1.34 (0.91, 1.98)	
Body mass index					0.392
<25 kg/m^2^	125,772	1 (reference)	1.02 (0.91, 1.15)	1.50 (1.13, 1.99)	
≥25 kg/m^2^	258,661	1 (reference)	1.09 (1.03, 1.15)	7.54 (1.06, 53.57)	
Alcohol status					0.237
Never	12,603	1 (reference)	1.24 (1.02, 1.50)	3.41 (1.25, 9.29)	
Previous	13,005	1 (reference)	0.96 (0.78, 1.17)	2.10 (0.93, 4.72)	
Current	358,825	1 (reference)	1.08 (1.03, 1.14)	1.50 (1.10, 2.03)	
Smoking status					0.422
Never	208,546	1 (reference)	1.05 (0.97, 1.13)	2.14 (1.42, 3.22)	
Previous	135,600	1 (reference)	1.12 (1.04, 1.21)	1.53 (0.93, 2.50)	
Current	40,287	1 (reference)	1.06 (0.91, 1.23)	1.10 (0.63, 1.90)	
Physical inactivity					0.542
No	254,762	1 (reference)	1.11 (1.03, 1.19)	1.55 (1.08, 2.24)	
Yes	45,117	1 (reference)	1.12 (0.99, 1.27)	1.80 (0.93, 3.50)	
Diet pattern					0.745
Healthy	30,031	1 (reference)	1.13 (0.93, 1.38)	2.99 (0.74, 12.03)	
Intermediate	192,688	1 (reference)	1.10 (1.02, 1.18)	1.47 (0.91, 2.37)	
Poor	73,622	1 (reference)	1.04 (0.93, 1.17)	2.21 (1.36, 3.57)	
Hypertension					0.226
No	175,640	1 (reference)	1.02 (0.91, 1.14)	1.75 (1.13, 2.70)	
Yes	208,793	1 (reference)	1.10 (1.04, 1.16)	1.47 (1.03, 2.09)	
Hyperlipidemia					0.158
No	207,319	1 (reference)	1.01 (0.93, 1.10)	1.63 (1.16, 2.29)	
Yes	177,114	1 (reference)	1.12 (1.05, 1.20)	1.50 (0.94, 2.38)	
Diabetes mellitus					0.186
No	363,320	1 (reference)	1.06 (1.00, 1.12)	1.50 (1.12, 2.01)	
Yes	21,113	1 (reference)	1.20 (1.06, 1.35)	3.36 (1.50, 7.54)	
Heart failure					0.363
No	383,223	1 (reference)	1.08 (1.03, 1.14)	1.56 (1.17, 2.06)	
Yes	1210	1 (reference)	1.03 (0.72, 1.46)	2.57 (0.58, 11.33)	
Valvular heart disease					0.018
No	382,634	1 (reference)	1.08 (1.03, 1.14)	1.56 (1.18, 2.06)	
Yes	1799	1 (reference)	1.19 (0.85, 1.67)	15.66 (3.63, 67.58)	
Coronary heart disease					0.741
No	365,396	1 (reference)	1.07 (1.01, 1.13)	1.53 (1.13, 2.05)	
Yes	19,037	1 (reference)	1.14 (1.01, 1.27)	2.05 (1.02, 4.12)	
COPD					0.782
No	377,163	1 (reference)	1.08 (1.02, 1.14)	1.57 (1.17, 2.12)	
Yes	7270	1 (reference)	1.15 (0.95, 1.38)	1.66 (0.82, 3.37)	
Autoimmune diseases					0.219
No	374,058	1 (reference)	1.08 (1.02, 1.14)	1.47 (1.08, 1.99)	
Yes	10,375	1 (reference)	1.13 (0.97, 1.33)	2.72 (1.43, 5.14)	
Hyperthyroidism					0.146
No	380,412	1 (reference)	1.08 (1.02, 1.14)	1.63 (1.24, 2.14)	
Yes	4021	1 (reference)	1.34 (0.91, 1.97)	0.00 (0.00, Inf)	

*Note*: Data were presented as hazard ratio (95% confidence interval). The Cox regression model was adjusted for age, gender, education level, Townsend deprivation index, body mass index, the status of drinking and smoking, physical inactivity, dietary pattern, laboratory tests (C‐reactive protein, alanine aminotransferase, creatinine, and albumin), comorbidities (heart failure, valvular heart disease, coronary heart disease, COPD, autoimmune diseases, hyperthyroidism, diabetes, hypertension, and hyperlipidemia), and major surgeries unless the covariate was used for stratification.

Abbreviation: COPD, chronic obstructive pulmonary disease.

### 3.4 Association between sarcopenia and AF incidence under different genetic backgrounds

**FIGURE 3:**
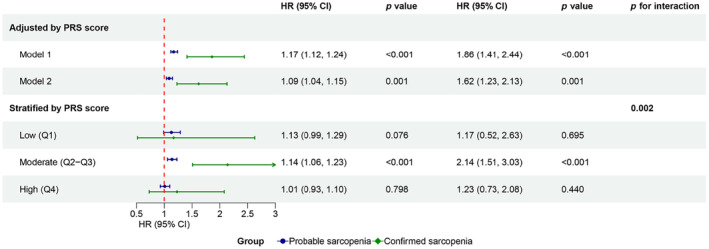
Association between sarcopenia and atrial fibrillation incidence in Cox regression models adjusted for PRS or stratified by PRS. Model 1 was adjusted for age, gender, education level, Townsend deprivation index, body mass index, and PRS. Based on model 1, model 2 was further adjusted for the status of drinking and smoking, physical inactivity, dietary pattern, laboratory tests (C‐reactive protein, alanine aminotransferase, creatinine, and albumin), comorbidities (heart failure, valvular heart disease, coronary heart disease, chronic obstructive pulmonary disease, autoimmune diseases, hyperthyroidism, diabetes, hypertension, and hyperlipidemia), and major surgeries. For stratification analysis, the Cox regression model adjusted for all covariates in model 2, except PRS. PRS, polygenic risk score.

As shown in Figure S9, the risk of developing AF increased with continuous PRS or PRS quartiles. No appreciable changes were found in the risk of AF incidence after adding PRS as an additional covariate into the multivariate models 1 and 2 (Figure 3). However, in the stratification analysis of PRS, the association of sarcopenia with a high incidence of AF was only presented in participants with moderate genetic risk for AF (Figure 3, *p* for interaction = 0.002). Similar results were noted for exposures of handgrip strength or muscle mass (Figure S10).

## 4 DISCUSSION

In the present study, we found that Caucasian participants with probable sarcopenia or confirmed sarcopenia were associated with a high long‐term risk of AF incidence after multivariate adjustment. The association remained robust in multiple sensitivity analyses. Additionally, the presence of low handgrip strength or low muscle mass alone was also associated with a high incidence of AF. Our findings were similar to those of previous cross‐sectional studies (Shim et al., 2023; Xia et al., 2021) and challenged the conclusion of a Korean prospective study that neither sarcopenia nor its components were associated with incident AF (Shim et al., 2023). We noted that the Korean cohort had limitations such as too short follow‐up time, limited sample size, too few confirmed cases of AF, and only including participants older than 70 years, which may hinder researchers from obtaining reliable results. To our knowledge, we have confirmed the longitudinal relationship between sarcopenia and AF for the first time, which may have a positive impact on related basic research and clinical practice in the future.

The underlying mechanism of sarcopenia affecting AF is complicated, with a possible crosstalk of obesity. In the previous study, compared with the control group, AF patients exhibited a decreased proportion of skeletal muscle but increased body mass index, fat weight, and waist circumference (Anaszewicz et al., 2019). Xia et al. (2021) found that concomitant low muscle mass and obesity/overweight rather than low muscle mass alone were associated with a notably higher risk of AF. With regard to our data, although sarcopenia was still independently associated with a high incidence of AF after adjusting for body mass index, the HR of new‐onset AF in the subgroup with body mass index ≥25 was about five times that in the subgroup with body mass index <25 among participants with confirmed sarcopenia. Mechanistically, sarcopenia and obesity may have a joint effect on AF. On the one hand, sarcopenia caused mitochondrial dysfunction and chronic inflammation in skeletal muscle to promote oxidative stress injury of cardiomyocytes, which further aggravated the damage of lipotoxicity on cardiomyocytes (Boengler et al., 2017; Li et al., 2019; Nattel, 2017). On the other hand, both sarcopenia and obesity were associated with impaired left ventricular diastolic function, resulting in left atrial overload (Iwasaki et al., 2012; Ko et al., 2018). These injuries ultimately cause profibrillatory remodeling of the left atrium to induce AF.

In the subgroup analysis, we found that there were differences in age, gender, the comorbidity of valvular heart disease, and genetic predisposition of AF risk based on sarcopenia. Epidemiologically, patients with AF are more likely to be older and male (Hindricks et al., 2021). Similarly, the incidence of sarcopenia increases with age (Cruz‐Jentoft et al., 2019). Muscle mass in men declines at a higher rate each year than that in women (Kohara et al., 2017). However, our data demonstrated that younger and female participants with sarcopenia were associated with a higher incidence of AF. Those with low epidemiological risk of AF were more likely to suffer from AF due to sarcopenia, suggesting that we should provide this population with more protective measures. Valvular heart disease is a definite cause of secondary AF (Hindricks et al., 2021). We found that the increased AF risk due to sarcopenia was substantially higher in participants with valvular heart disease than in those without this comorbidity. It may be explained that sarcopenia could promote cardiac remodeling (Boengler et al., 2017; Ko et al., 2018; Li et al., 2019) and then exert an additive effect on AF development on the basis of valvular heart disease. Importantly, according to our findings, it should also be heightened to prevent sarcopenia in patients with valvular heart disease. In addition, we found that sarcopenia was significantly associated with a high risk of AF only in participants with moderate PRS, which indicated that the comorbidity of sarcopenia did not exert an appreciable effect on the original genetic risk of AF. Since sarcopenia was still associated with high AF incidence after adjusting for PRS, we believed that differences in this subgroup were not enough to change the main conclusion of this study.

According to our findings, prevention of sarcopenia may help reduce the incidence of AF. Sarcopenia is strongly associated with developmental plasticity, so we should increase muscle in adolescence and delay muscle loss in middle age and old age (Sayer et al., 2008). Another effective measure to prevent sarcopenia is to identify patients with probable sarcopenia as soon as possible and initiate interventions. Low handgrip strength is the precursor of sarcopenia (Cruz‐Jentoft et al., 2019). This study showed that preventing further muscle mass loss could greatly reduce the HR of developing AF. A multimodal approach aimed at improving exercise capacity and energy supply was proposed to alleviate the wasting status of the human body such as sarcopenia (Bielecka‐Dabrowa et al., 2020). Both aerobic exercise and resistance exercise could effectively maintain muscle function and increase muscle mass in elderly people with pre‐sarcopenia, which may be attributed to the increase of skeletal muscle protein reserves and sensitivity to insulin (Cunha et al., 2012; Sáez de Asteasu et al., 2020; Vikberg et al., 2019). Nutritional supplementation, especially excessive protein intake, could further enhance the beneficial effects of physical activity (Malafarina et al., 2013; Morton et al., 2018).

The major strength of this study is that it is a prospective study exploring the longitudinal relationship between sarcopenia and AF, with a large sample size, long follow‐up time, and various sensitivity analyses. However, this study has some limitations. First, there was a lack of objective physical performance measurement in the assessment of sarcopenia, which did not allow us to include severe sarcopenia in the analysis. Second, the observational data used in this study were affected by confounding factors, although we adjusted for as many covariates as possible. Third, all covariates were obtained at the baseline survey. This study did not take into account the changes in these covariates during the follow‐up period, which may influence the risk of developing AF to some extent. Lastly, this population‐based cohort study had a selection bias for healthy volunteers. Volunteers with lower disease prevalence and incidence were more likely to be included in the study.

## 5 CONCLUSIONS

In conclusion, both probable and confirmed sarcopenia were associated with a high long‐term risk of AF in Caucasians. These findings provided new evidence for the longitudinal relationship between sarcopenia and AF and suggested that preventing or delaying sarcopenia may help reduce the disease burden of AF, especially for younger, female individuals and those with valvular heart disease.

## AUTHOR CONTRIBUTIONS

Lihuang Zha and Benhui Liang were responsible for the study's design, and were granted complete access to all the data. Yiyang Tang and Zhenghui Liu conducted the analyses of data. Yiyang Tang drafted this manuscript. Qin Chen, Mukamengjiang Juaiti, and Zaixin Yu revised this manuscript, and contributed to the interpretation of the findings. The final manuscript has received approval from all authors, and they confirm that the manuscript has not been previously published elsewhere and is not under consideration by any other journal.

## FUNDING INFORMATION

This work was supported by the National Natural Science Foundation of China under grants No.82070055 and No. 82100071, Hunan Provincial Natural Science Foundation of China under grants No.2022JJ30981 and No.2022JJ40769, and Fundamental Research Funds for the Central Universities of Central South University under the grants No.2023ZZTS0550.

## CONFLICT OF INTEREST STATEMENT

The authors declare that they have no competing interests.

## Supporting information


Appendix S1.

## Data Availability

The data pertinent to this study can be procured from the UK Biobank Resource (www.ukbiobank.ac.uk). It is important to note that an application, followed by formal approval, is necessary to access the data.
