# Supplementary material for: Association of sarcopenia with the long‐term risk of atrial fibrillation: A prospective cohort study
Source: Aging Cell. 2024 May 13;23(8):e14198. doi: 10.1111/acel.14198 (PMC11320353; doi:10.1111/acel.14198)
Supplement: Supplementary file 1 — Appendix S1. [file ACEL-23-e14198-s001.docx]

**
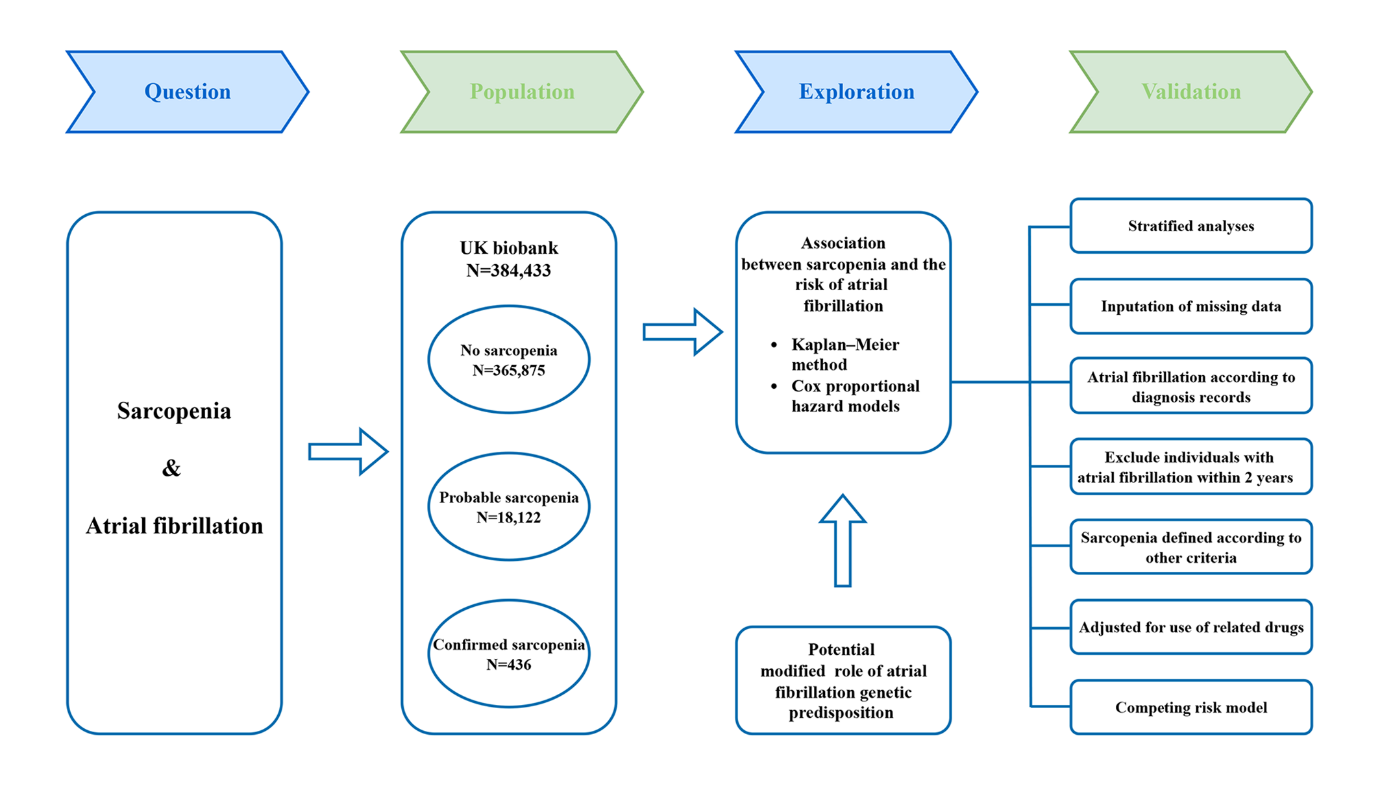
**

**Figure S1. Study design**

**
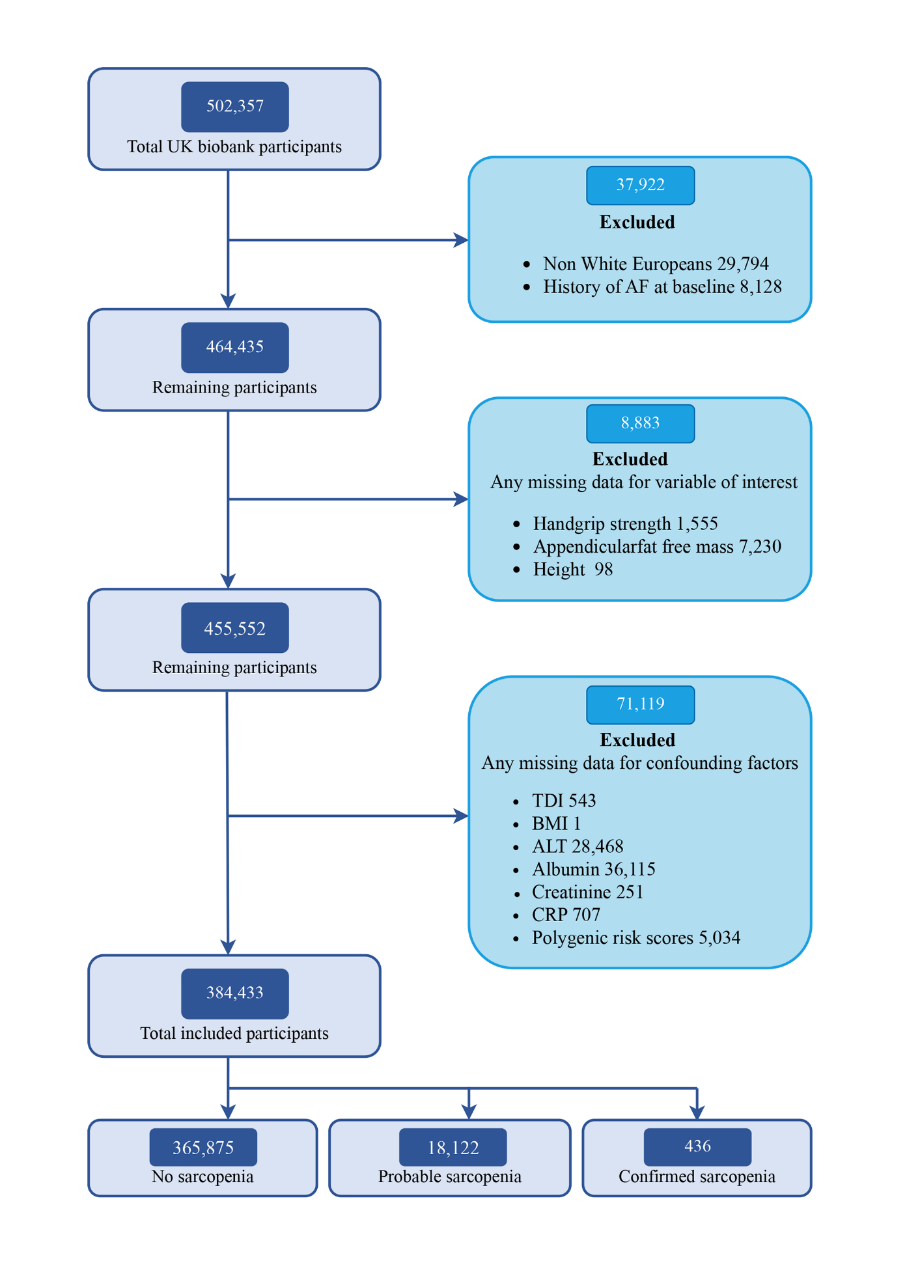
**

**Figure S2. Flowchart of inclusion and exclusion of participants**

**
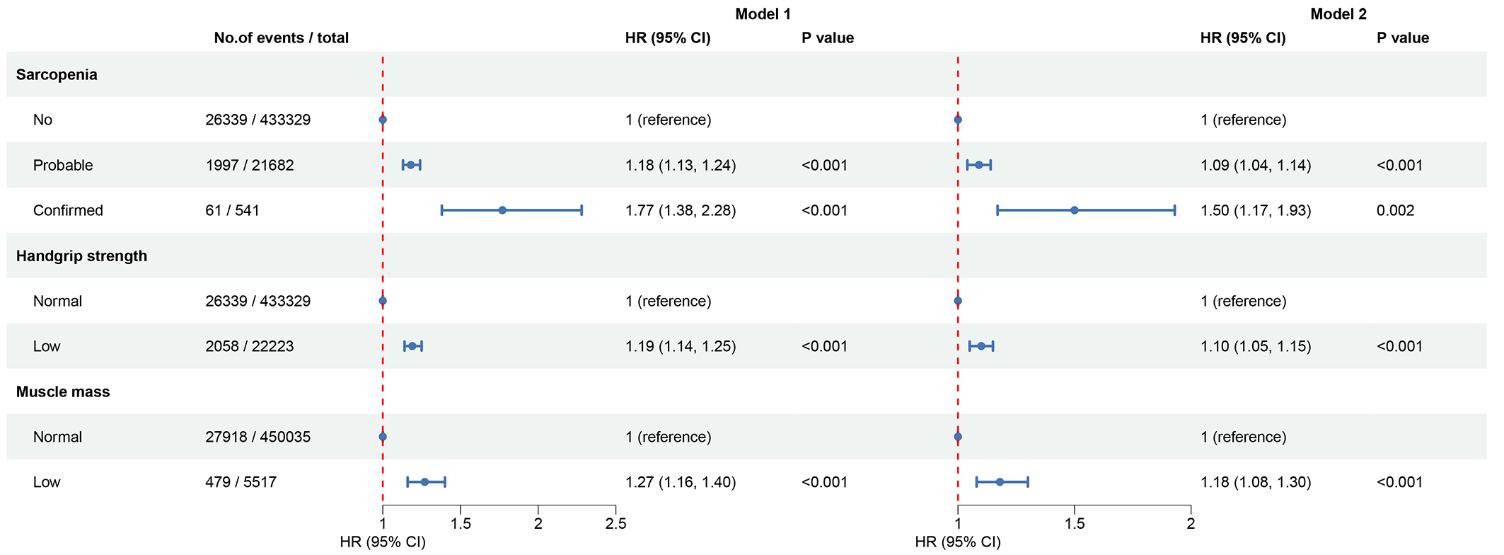
**

**Figure S3. Sensitivity analysis of the association of sarcopenia and its components with the incidence of atrial fibrillation after multiple imputations for missing values of covariates.**

Model 1 was adjusted for age, gender, education level, Townsend deprivation index, and body mass index. Based on model 1, model 2 was further adjusted for the status of drinking and smoking, physical inactivity, dietary pattern, laboratory tests (C-reactive protein, alanine aminotransferase, creatinine, and albumin), comorbidities (heart failure, valvular heart disease, coronary heart disease, chronic obstructive pulmonary disease, autoimmune diseases, hyperthyroidism, diabetes, hypertension, and hyperlipidemia), and major surgeries.

**
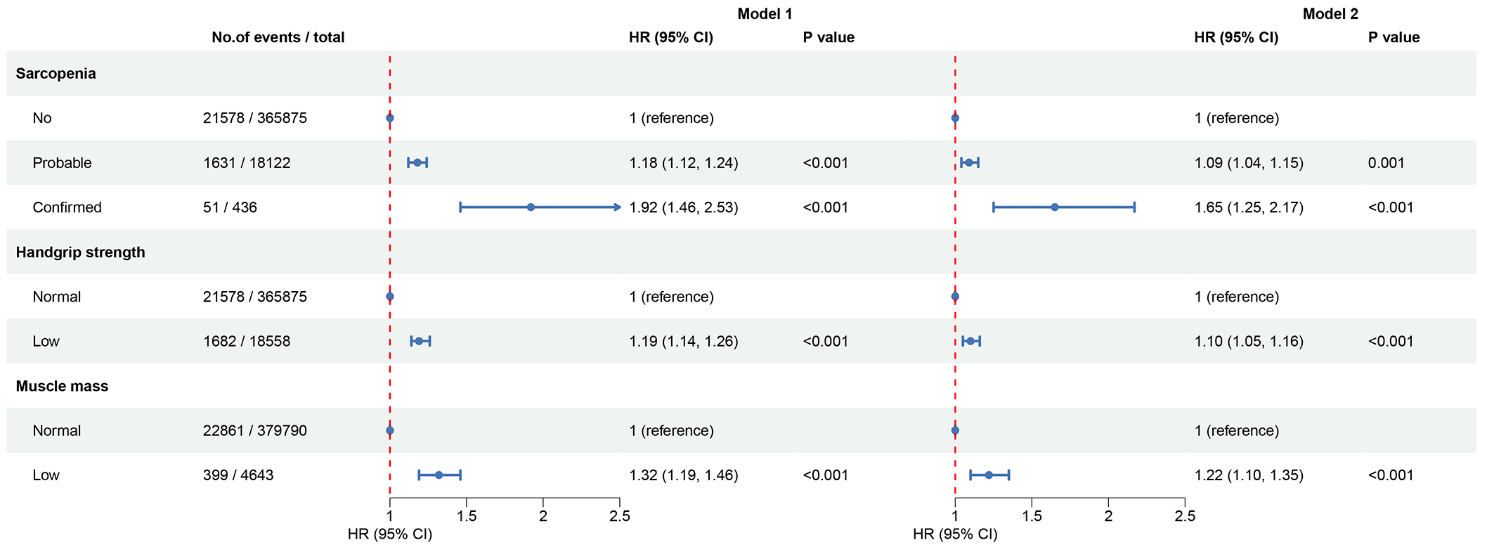
**

**Figure S4. Sensitivity analysis of the association of sarcopenia and its components with the incidence of atrial fibrillation by limiting the diagnosis of atrial fibrillation to hospital-recorded diagnosis.**

Model 1 was adjusted for age, gender, education level, Townsend deprivation index, and body mass index. Based on model 1, model 2 was further adjusted for the status of drinking and smoking, physical inactivity, dietary pattern, laboratory tests (C-reactive protein, alanine aminotransferase, creatinine, and albumin), comorbidities (heart failure, valvular heart disease, coronary heart disease, chronic obstructive pulmonary disease, autoimmune diseases, hyperthyroidism, diabetes, hypertension, and hyperlipidemia), and major surgeries.

**
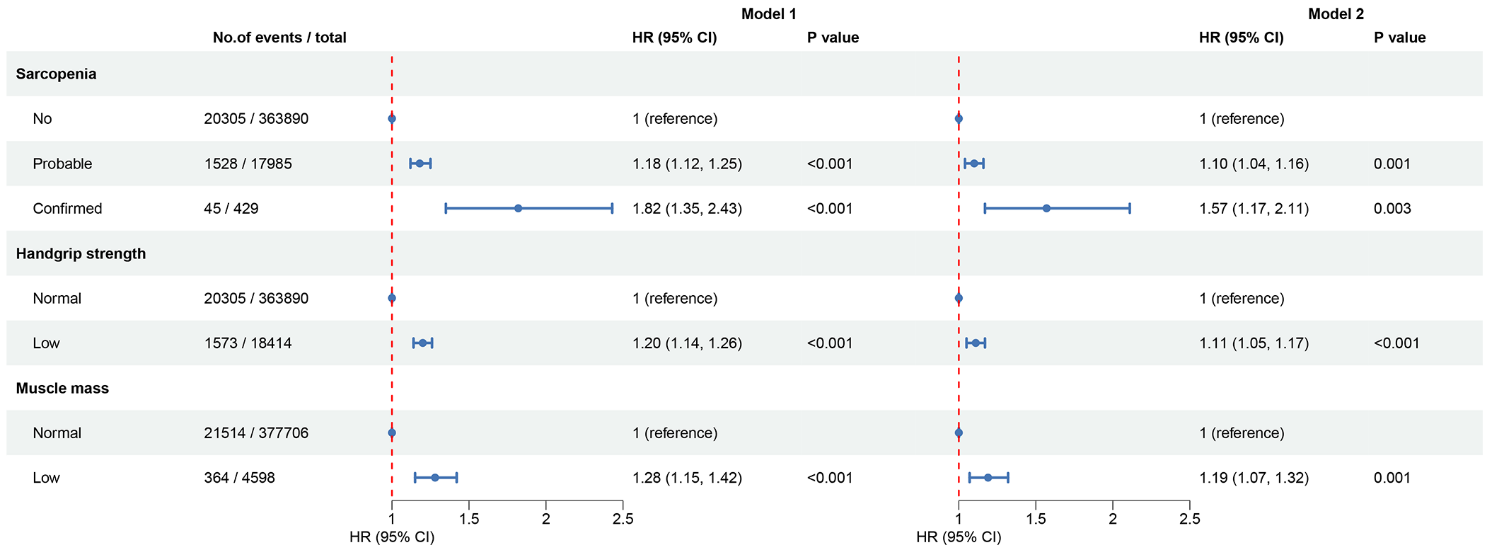
**

**Figure S5. Sensitivity analysis of the association of sarcopenia and its components with the incidence of atrial fibrillation by excluding participants who experienced new-onset atrial fibrillation within the first two years of the follow-up.**

Model 1 was adjusted for age, gender, education level, Townsend deprivation index, and body mass index. Based on model 1, model 2 was further adjusted for the status of drinking and smoking, physical inactivity, dietary pattern, laboratory tests (C-reactive protein, alanine aminotransferase, creatinine, and albumin), comorbidities (heart failure, valvular heart disease, coronary heart disease, chronic obstructive pulmonary disease, autoimmune diseases, hyperthyroidism, diabetes, hypertension, and hyperlipidemia), and major surgeries.

**
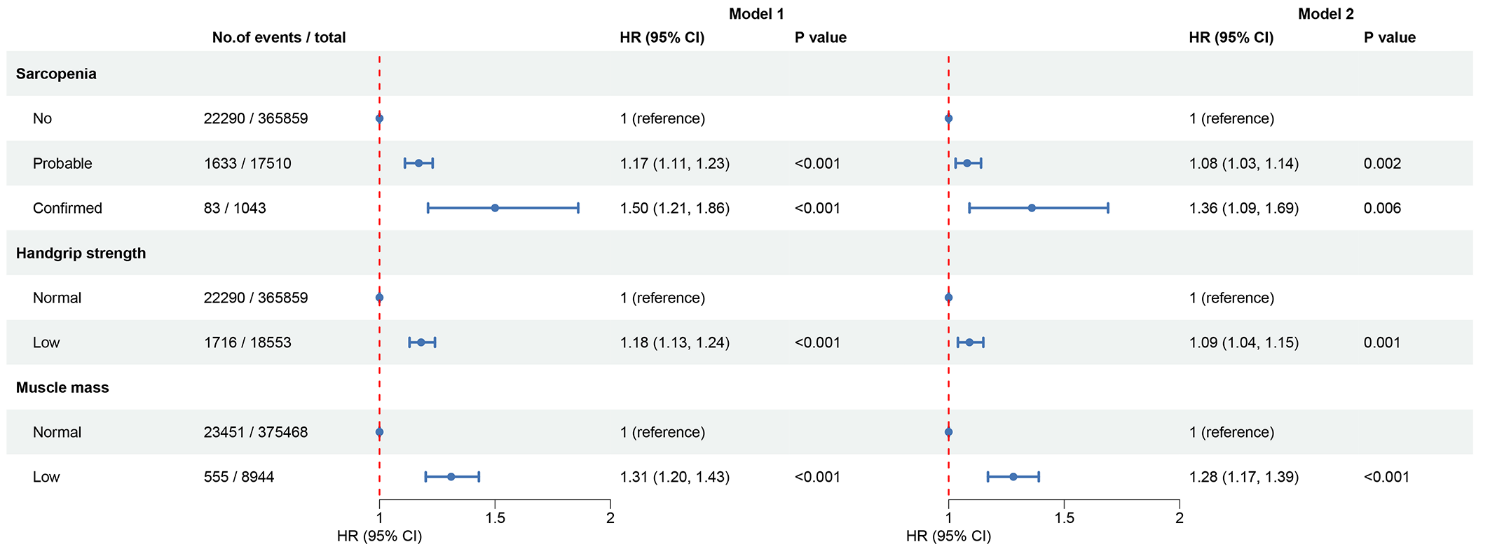
**

**Figure S6. Sensitivity analysis of the association of sarcopenia and its components with the incidence of atrial fibrillation by using another sarcopenia assessment criterion.**

Model 1 was adjusted for age, gender, education level, Townsend deprivation index, and body mass index. Based on model 1, model 2 was further adjusted for the status of drinking and smoking, physical inactivity, dietary pattern, laboratory tests (C-reactive protein, alanine aminotransferase, creatinine, and albumin), comorbidities (heart failure, valvular heart disease, coronary heart disease, chronic obstructive pulmonary disease, autoimmune diseases, hyperthyroidism, diabetes, hypertension, and hyperlipidemia), and major surgeries.

**
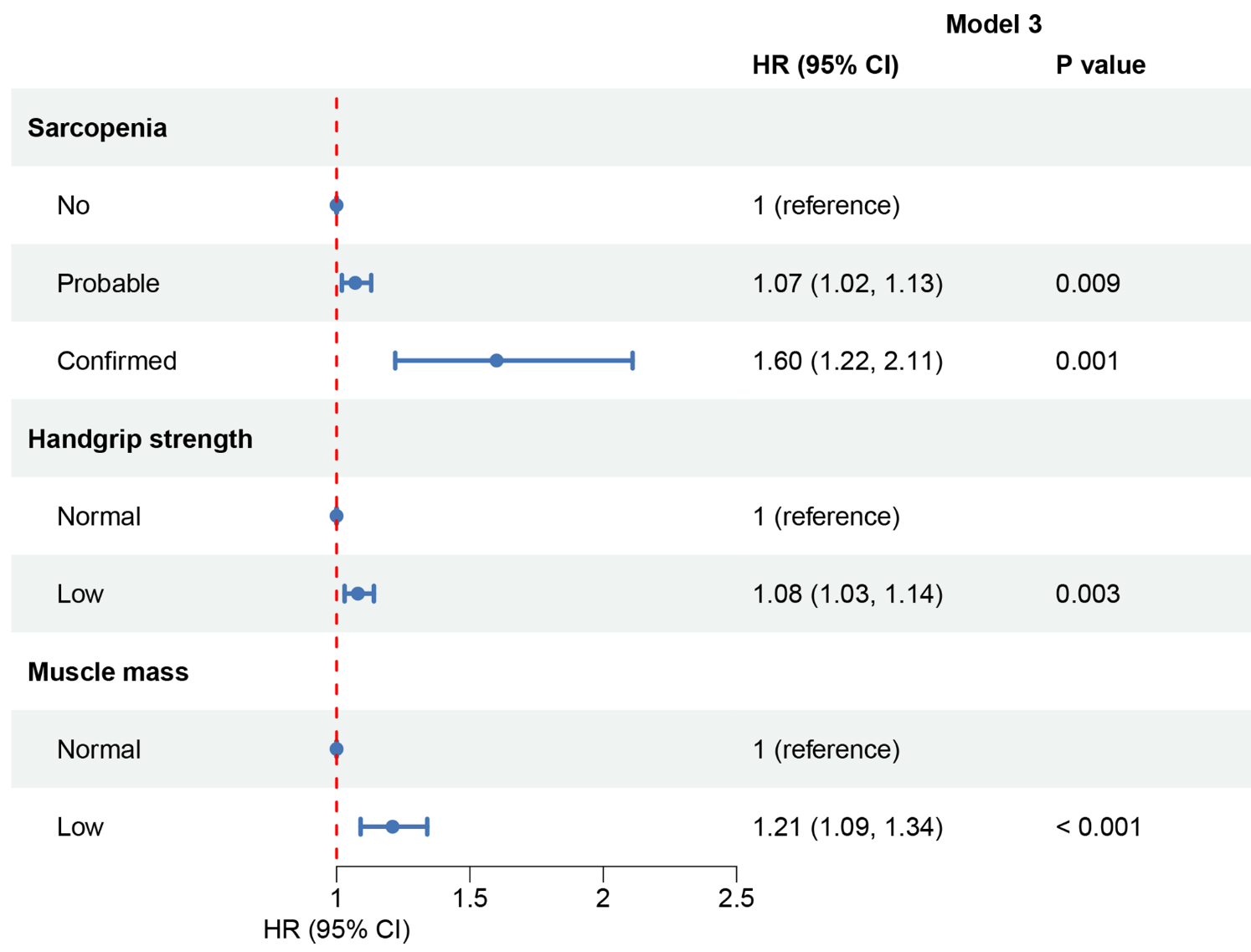
**

**Figure S7. Sensitivity analysis of the association of sarcopenia and its components with the incidence of atrial fibrillation by further adjusting for medication intake.**

Model 3 was adjusted for age, gender, education level, Townsend deprivation index, body mass index, status of drinking and smoking, physical inactivity, dietary pattern, laboratory tests (C-reactive protein, alanine aminotransferase, creatinine, and albumin), comorbidities (heart failure, valvular heart disease, coronary heart disease, chronic obstructive pulmonary disease, autoimmune diseases, hyperthyroidism, diabetes, hypertension, and hyperlipidemia), major surgeries, and medication intake (antiplatelet, antihypertensive, antidiabetic, and cholesterol-lowering drugs).

**
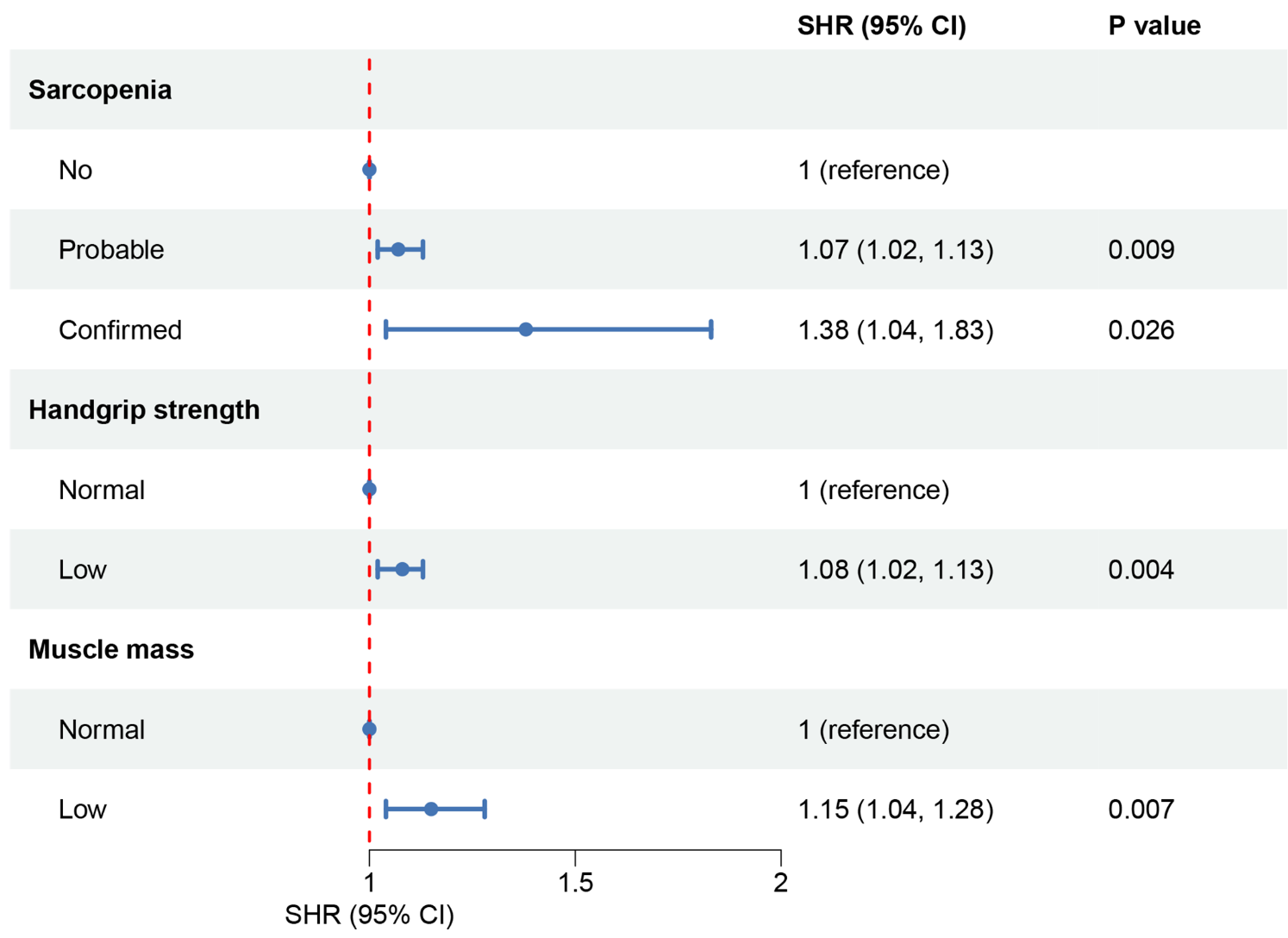
**

**Figure S8. Sensitivity analysis of the association of sarcopenia and its components with atrial fibrillation incidence by incorporating a competing risk model.**

The model was adjusted for age, gender, education level, Townsend deprivation index, body mass index, the status of drinking and smoking, physical inactivity, dietary pattern, laboratory tests (C-reactive protein, alanine aminotransferase, creatinine, and albumin), comorbidities (heart failure, valvular heart disease, coronary heart disease, chronic obstructive pulmonary disease, autoimmune diseases, hyperthyroidism, diabetes, hypertension, and hyperlipidemia), and major surgeries. SHR, sub-distribution hazard ratio.

**
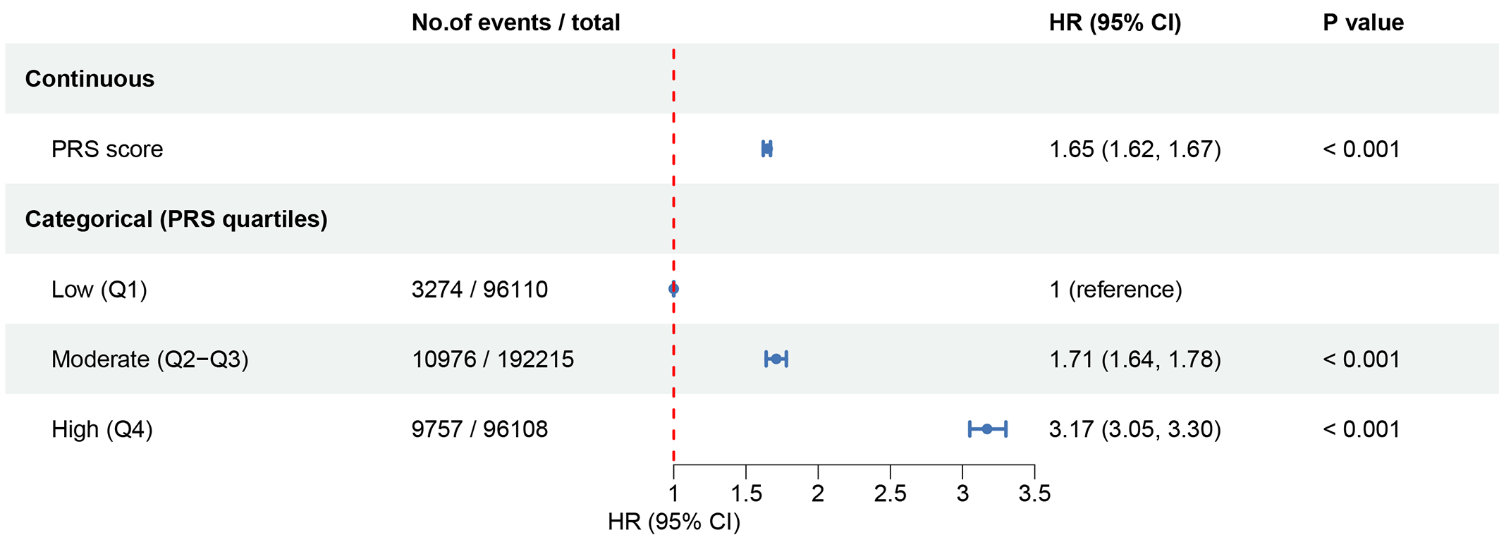
**

**Figure S9. Association between the PRS of atrial fibrillation with atrial fibrillation incidence examined by Cox proportional hazards regression analysis.**

The model was adjusted for age, gender, education level, Townsend deprivation index, body mass index, the status of drinking and smoking, physical inactivity, dietary pattern, laboratory tests (C-reactive protein, alanine aminotransferase, creatinine, and albumin), comorbidities (heart failure, valvular heart disease, coronary heart disease, chronic obstructive pulmonary disease, autoimmune diseases, hyperthyroidism, diabetes, hypertension, and hyperlipidemia), and major surgeries. PRS, polygenic risk score.

**
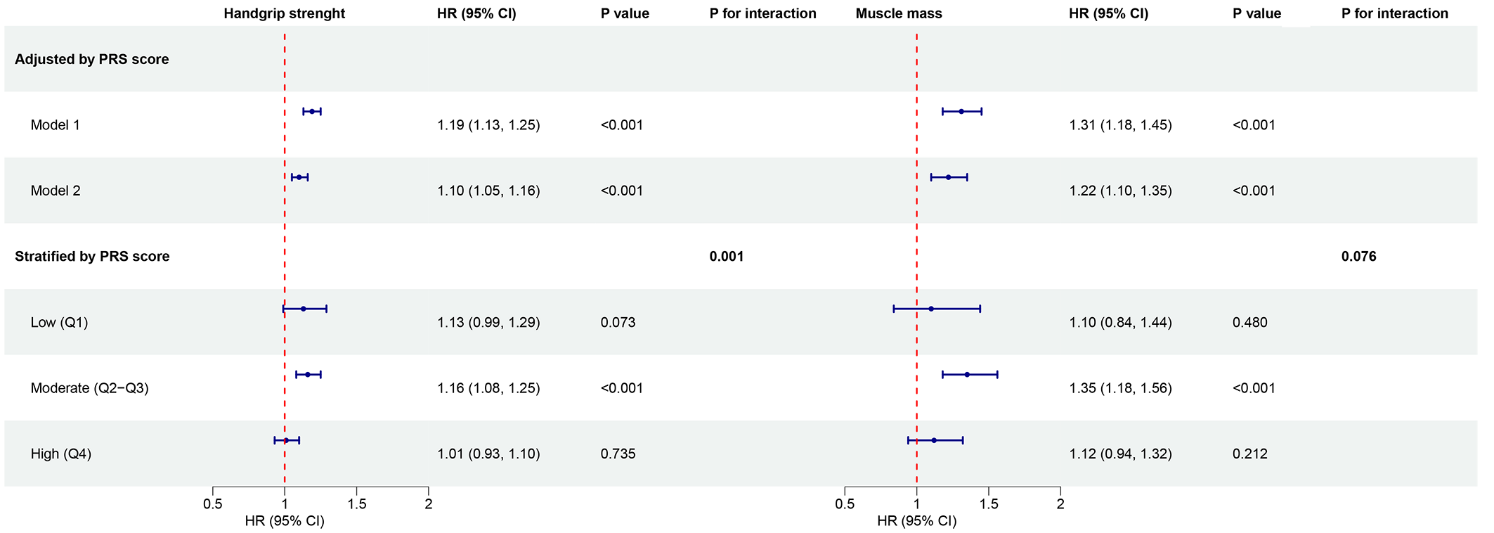
**

**Figure S10. Association of handgrip strength and muscle mass with atrial fibrillation incidence in Cox regression models adjusted for PRS or stratified by PRS.**

Model 1 was adjusted for age, gender, education level, Townsend deprivation index, body mass index, and PRS. Based on model 1, model 2 was further adjusted for the status of drinking and smoking, physical inactivity, dietary pattern, laboratory tests (C-reactive protein, alanine aminotransferase, creatinine, and albumin), comorbidities (heart failure, valvular heart disease, coronary heart disease, chronic obstructive pulmonary disease, autoimmune diseases, hyperthyroidism, diabetes, hypertension, and hyperlipidemia), and major surgeries. For subgroup analysis, the Cox regression model adjusted for all covariates in model 2, except PRS. PRS, polygenic risk score.

**Table S1.** **Codes of variables in the UK Biobank.**

|  | Filed ID | Codes |
| --- | --- | --- |
| Age | 21022 |  |
| Sex | 31 |  |
| Education | 6138 |  |
| Townsend deprivation index | 22189 |  |
| Body mass index | 21001 |  |
| Drinking status | 20117 |  |
| Smoking status | 20116 |  |
| Physical activity | 22040 |  |
| C-reactive protein | 30710 |  |
| Alanine aminotransferase | 30650 |  |
| Albumin | 30600 |  |
| Creatinine | 30700 |  |
| Polygenic risk score | 26212 |  |
| Atrial fibrillation | 131350-131351 | I48 |
| Heart failure | 131354-131355 | I50 |
| Valvular heart disease | 131322-131329  - | I34-I37 |
| Coronary heart disease | 6150, 131298-131307 | 1-2, I21-25 |
| Chronic obstructive pulmonary disease | 131490-131493 | J43–J44 |
| Autoimmune diseases | 131626-131629, 131848-131851, 131890-131897 | K50-K51, M05-M06, M30-33 |
| Hyperthyroidism | 130700-130701 | E5 |
| Hypertension | 131286-131295 | I10-I13, I15 |
| Diabetes mellitus | 130706-130715 | E10-E14 |
| Major surgeries | 2415, 2844 | 1 |
| Antiplatelet drugs | 6154, 20003 | 1, 1140861806, 1140864860, 1140868226, 1140868282, 1140872040, 1140882108, 1140882190, 1140882268, 1140882392, 1141163138, 1141164044, 1141167844, 1141168318, 1140861778, 1141181150 |
| Antihypertensive drugs | 6177, 6153, 20003 | 2, 1140860696, 1140860728, 1140860738, 1140860750, 1140860752, 1140860764, 1140860790, 1140860806, 1140860882, 1140860904, 1140864952, 1140888552, 1140888556, 1140888560, 1140923712, 1141153328, 1141164148, 1141165470, 1141180592, 1140916356, 1141145660, 1141151016, 1141152998, 1141156836, 1141166006, 1141171336, 1141172682, 1141187788, 1141201038, 1140860426, 1140861088, 1140861190, 1140861276, 1140872568, 1140879802, 1140879810, 1140888646, 1140923572, 1140928226, 1141153026, 1141165470, 1140888510, 1141153328, 1140860332, 1140860404, 1140860422, 1140860562, 1140864950, 1140926778, 1140879806, 1140866244, 1140860308 |
| Antidiabetic drugs | 6177, 6153, 20003 | 3, 1140883066, 1140884600, 1141189090, 1140857494, 1140874646, 1140874650, 1140874658, 1140874718, 1140874744, 1141152590, 1141153254, 1141157284, 1141168660, 1141171646, 1141173882, 1141177600, 1140868902 |
| Cholesterol-lowering drugs | 6177, 6153, 20003 | 1, 1140861924, 1140861944, 1140861954, 1140862026, 1141157260, 1140861892, 1141192736, 1140861958, 1140888594, 1140888648, 1140910632, 1140910654, 1141146234, 1141192410 |

**Table S2.** **The original and adapted assessment criteria of sarcopenia.**

| Component | Original criteria | |  | UK Biobank | |
| --- | --- | --- | --- | --- | --- |
|  | Women | Men |  | Women | Men |
| Sarcopenia ^a^ |  |  |  |  |  |
| Low handgrip strength | < 16 kg | < 27 kg |  | < 16 kg | < 27 kg |
| Low muscle mass | SMM ^b^/height^2^ < 5.5 kg/m^2^ | SMM/height^2^ < 7.0 kg/m^2^ |  | ALST ^c^/height^2^ < 5.30 kg/m^2^ | ALST/height^2^ < 6.95 kg/m^2^ |
| Low physical performance | Gait speed < 0.8 m/s | |  | Self-reported: ‘How would you describe your usual walking pace?’  Response: slow and other (average, brisk, none of the above) | |

^a^ The assessment of sarcopenia followed the guidelines of the European Working Group on Sarcopenia in Older People.

^b^ The whole-body SMM was calculated using the Janssen equation.

^c^ The estimation of ALST conformed to the equation: ALST (kg) = (0.958 × [AFFM(kg)]) − (0.166 × S) − 0.308. In this equation, 'S' is assigned a value of 0 for women and 1 for men. This formula was initially developed using data from participants (n=4350) in the UK Biobank who underwent dual x-ray absorptiometry body composition scans. Furthermore, the sex-specific cut-off points for low ALST/height², specific to the UK Biobank cohort, were derived from participants < 45 years old. It was used as the reference based on two standard deviations below the sex-specific mean.

Abbreviation: SMM, skeletal muscle mass; ALST, appendicular lean soft tissue; AFFM, appendicular fat-free mass.

**Table S3. Assessment of a healthy diet in the UK Biobank.**

| **Variables** | **Filed ID** | **Descriptions** | **Criteria of a healthy diet** |
| --- | --- | --- | --- |
| vegetables | 1289 | Cooked vegetables | ≥3 servings/day  1 serving: 3 tablespoons of salad/raw/cooked vegetables |
|  | 1299 | Salad/raw vegetables |  |
| Fruits | 1309 | Fresh fruits | ≥3 servings/day  1 serving: 1 piece of fresh fruits or 5 pieces of dried fruits |
|  | 1319 | Dried fruits |  |
| Fish | 1329 | Oily fish | ≥2 servings/week  1 serving: eating oily fish or non-oily fish once a week |
|  | 1339 | Non-oily fish |  |
| Processed meats | 1349 | Processed meats | ≤1 servings/week  1 serving: eating processed meats once a week |
| Unprocessed red meats | 1369 | Beef | ≤2 servings/week  1 serving: eating beef, lamb/mutton or pork once a week |
|  | 1379 | Lamb/mutton |  |
|  | 1389 | Pork |  |
| Whole grains | 1448 | Bread type | ≥3 servings/day  1 serving: 1 slice of wholemeal/wholegrain bread or 1 bowl of bran/oat/muesli cereal |
|  | 1468 | Cereal type |  |
|  | 1438 | Bread intake |  |
|  | 1458 | Cereal intake |  |
| Refined grains | 1448 | Bread type | ≤1.5 servings/day  1 serving: 1 slice of white/brown/other bread or 1 bowl of biscuit/other cereal |
|  | 1468 | Cereal type |  |
|  | 1438 | Bread intake |  |
|  | 1458 | Cereal intake |  |

**Table S4. Subgroup analysis of the association of handgrip strength and muscle mass with the incidence of atrial fibrillation.**

| **Subgroups** | **N** | **Handgrip strength** | | |  | **Muscle mass** | | |
| --- | --- | --- | --- | --- | --- | --- | --- | --- |
|  |  | **Normal** | **Low** | ***p* for interaction** |  | **Normal** | **Low** | ***p* for interaction** |
| **Sex** |  |  |  | 0.514 |  |  |  | 0.041 |
| Women | 208923 | 1 (reference) | 1.06 (0.99, 1.14) |  |  | 1 (reference) | 1.69 (1.14, 2.51) |  |
| Men | 175510 | 1 (reference) | 1.11 (1.03, 1.19) |  |  | 1 (reference) | 1.26 (1.13, 1.40) |  |
| **Age** |  |  |  | 0.001 |  |  |  | 0.121 |
| <65 | 310838 | 1 (reference) | 1.23 (1.14, 1.32) |  |  | 1 (reference) | 1.42 (1.22, 1.65) |  |
| ≥65 | 73595 | 1 (reference) | 1.10 (1.03, 1.18) |  |  | 1 (reference) | 1.22 (1.07, 1.40) |  |
| **TDI** |  |  |  | 0.966 |  |  |  | 0.376 |
| Low | 77441 | 1 (reference) | 1.09 (0.96, 1.25) |  |  | 1 (reference) | 1.00 (0.77, 1.28) |  |
| Moderate | 230200 | 1 (reference) | 1.09 (1.02, 1.16) |  |  | 1 (reference) | 1.25 (1.09, 1.43) |  |
| High | 76792 | 1 (reference) | 1.10 (1.01, 1.21) |  |  | 1 (reference) | 1.27 (1.05, 1.54) |  |
| **Education** |  |  |  | 0.373 |  |  |  | 0.493 |
| College | 54662 | 1 (reference) | 1.14 (0.97, 1.34) |  |  | 1 (reference) | 0.92 (0.70, 1.22) |  |
| High school | 17176 | 1 (reference) | 1.24 (0.96, 1.61) |  |  | 1 (reference) | 1.20 (0.73, 1.97) |  |
| Middle school | 178177 | 1 (reference) | 1.02 (0.93, 1.12) |  |  | 1 (reference) | 1.29 (1.09, 1.53) |  |
| Others | 134418 | 1 (reference) | 1.11 (1.04, 1.18) |  |  | 1 (reference) | 1.23 (1.07, 1.43) |  |
| **BMI** |  |  |  | 0.574 |  |  |  | 0.255 |
| <25 Kg/m^2^ | 125772 | 1 (reference) | 1.06 (0.95, 1.18) |  |  | 1 (reference) | 1.27 (1.14, 1.41) |  |
| ≥25 Kg/m^2^ | 258661 | 1 (reference) | 1.09 (1.03, 1.16) |  |  | 1 (reference) | 2.01 (0.75, 5.35) |  |
| **Alcohol status** |  |  |  | 0.181 |  |  |  | 0.704 |
| Never | 12603 | 1 (reference) | 1.26 (1.04, 1.53) |  |  | 1 (reference) | 1.18 (0.68, 2.05) |  |
| Previous | 13005 | 1 (reference) | 0.98 (0.81, 1.20) |  |  | 1 (reference) | 1.40 (0.94, 2.09) |  |
| Current | 358825 | 1 (reference) | 1.09 (1.03, 1.15) |  |  | 1 (reference) | 1.20 (1.08, 1.33) |  |
| **Smoking status** |  |  |  | 0.838 |  |  |  | 0.736 |
| Never | 208546 | 1 (reference) | 1.06 (0.99, 1.15) |  |  | 1 (reference) | 1.24 (1.06, 1.44) |  |
| Previous | 135600 | 1 (reference) | 1.13 (1.04, 1.21) |  |  | 1 (reference) | 1.19 (1.01, 1.42) |  |
| Current | 40287 | 1 (reference) | 1.06 (0.91, 1.22) |  |  | 1 (reference) | 1.11 (0.90, 1.37) |  |
| **Physical inactivity** |  |  |  | 0.214 |  |  |  | 0.376 |
| No | 254762 | 1 (reference) | 1.12 (1.04, 1.20) |  |  | 1 (reference) | 1.24 (1.10, 1.40) |  |
| Yes | 45117 | 1 (reference) | 1.13 (1.00, 1.28) |  |  | 1 (reference) | 1.20 (0.88, 1.63) |  |
| **Dietary pattern** |  |  |  | 0.862 |  |  |  | 0.659 |
| Healthy | 30031 | 1 (reference) | 1.15 (0.94, 1.39) |  |  | 1 (reference) | 1.49 (1.00, 2.22) |  |
| Intermediate | 192688 | 1 (reference) | 1.10 (1.02, 1.19) |  |  | 1 (reference) | 1.21 (1.04, 1.41) |  |
| Poor | 73622 | 1 (reference) | 1.07 (0.96, 1.20) |  |  | 1 (reference) | 1.24 (1.00, 1.53) |  |
| **Hypertension** |  |  |  | 0.359 |  |  |  | 0.037 |
| No | 175640 | 1 (reference) | 1.04 (0.93, 1.16) |  |  | 1 (reference) | 1.22 (1.03, 1.44) |  |
| Yes | 208793 | 1 (reference) | 1.10 (1.04, 1.17) |  |  | 1 (reference) | 1.15 (1.01, 1.30) |  |
| **Hyperlipidemia** |  |  |  | 0.11 |  |  |  | 0.006 |
| No | 207319 | 1 (reference) | 1.03 (0.95, 1.12) |  |  | 1 (reference) | 1.31 (1.15, 1.49) |  |
| Yes | 177114 | 1 (reference) | 1.13 (1.06, 1.20) |  |  | 1 (reference) | 1.04 (0.89, 1.23) |  |
| **Diabetes mellitus** |  |  |  | 0.156 |  |  |  | 0.778 |
| No | 363320 | 1 (reference) | 1.07 (1.01, 1.13) |  |  | 1 (reference) | 1.18 (1.06, 1.31) |  |
| Yes | 21113 | 1 (reference) | 1.21 (1.08, 1.36) |  |  | 1 (reference) | 1.50 (0.94, 2.40) |  |
| **Heart failure** |  |  |  | 0.719 |  |  |  | 0.274 |
| No | 383223 | 1 (reference) | 1.09 (1.04, 1.15) |  |  | 1 (reference) | 1.19 (1.08, 1.32) |  |
| Yes | 1210 | 1 (reference) | 1.05 (0.75, 1.49) |  |  | 1 (reference) | 1.97 (0.97, 4.00) |  |
| **Valvular heart disease** |  |  |  | 0.872 |  |  |  | 0.055 |
| No | 382634 | 1 (reference) | 1.09 (1.03, 1.15) |  |  | 1 (reference) | 1.19 (1.08, 1.32) |  |
| Yes | 1799 | 1 (reference) | 1.26 (0.91, 1.76) |  |  | 1 (reference) | 1.89 (1.06, 3.35) |  |
| **Coronary heart disease** |  |  |  | 0.732 |  |  |  | 0.590 |
| No | 365396 | 1 (reference) | 1.08 (1.02, 1.14) |  |  | 1 (reference) | 1.18 (1.06, 1.32) |  |
| Yes | 19037 | 1 (reference) | 1.15 (1.02, 1.29) |  |  | 1 (reference) | 1.27 (0.96, 1.67) |  |
| **COPD** |  |  |  | 0.638 |  |  |  | 0.115 |
| No | 377163 | 1 (reference) | 1.09 (1.03, 1.15) |  |  | 1 (reference) | 1.18 (1.06, 1.32) |  |
| Yes | 7270 | 1 (reference) | 1.17 (0.97, 1.40) |  |  | 1 (reference) | 1.21 (0.89, 1.64) |  |
| **Autoimmune diseases** |  |  |  | 0.723 |  |  |  | 0.017 |
| No | 374058 | 1 (reference) | 1.09 (1.03, 1.15) |  |  | 1 (reference) | 1.17 (1.06, 1.30) |  |
| Yes | 10375 | 1 (reference) | 1.16 (0.99, 1.36) |  |  | 1 (reference) | 1.88 (1.24, 2.83) |  |
| **Hyperthyroidism** |  |  |  | 0.101 |  |  |  | 0.475 |
| No | 380412 | 1 (reference) | 1.09 (1.03, 1.15) |  |  | 1 (reference) | 1.20 (1.09, 1.33) |  |
| Yes | 4021 | 1 (reference) | 1.32 (0.90, 1.93) |  |  | 1 (reference) | 2.20 (0.67, 7.21) |  |

Data were presented as hazard ratio (95% confidence interval). The Cox regression model was adjusted for age, gender, education level, TDI, BMI, the status of drinking and smoking, physical inactivity, dietary pattern, laboratory tests (C-reactive protein, alanine aminotransferase, creatinine, and albumin), comorbidities (heart failure, valvular heart disease, coronary heart disease, COPD, autoimmune diseases, hyperthyroidism, diabetes, hypertension, and hyperlipidemia), and major surgeries unless the covariate was used for stratification. BMI, body mass index; TDI, Townsend deprivation index; COPD, chronic obstructive pulmonary disease.
